# Genome-wide copy number variation analysis in a Chinese autism spectrum disorder cohort

**DOI:** 10.1038/srep44155

**Published:** 2017-03-10

**Authors:** Hui Guo, Yu Peng, Zhengmao Hu, Ying Li, Guanglei Xun, Jianjun Ou, Liangdan Sun, Zhimin Xiong, Yanling Liu, Tianyun Wang, Jingjing Chen, Lu Xia, Ting Bai, Yidong Shen, Qi Tian, Yiqiao Hu, Lu Shen, Rongjuan Zhao, Xuejun Zhang, Fengyu Zhang, Jingping Zhao, Xiaobing Zou, Kun Xia

**Affiliations:** 1 The State Key Laboratory of Medical Genetics, School of Life Sciences, Central South University, Changsha, Hunan, China; 2 Mental Health Institute, the Second Xiangya Hospital, Central South University, Changsha, Hunan, China; 3 Mental Health Center of Shandong Province, Jinan, Shandong, China; 4 State Key Laboratory Incubation Base of Dermatology, Hefei, Anhui, China; 5 The Third Xiangya Hospital, Central South University, Changsha, Hunan, China; 6 The Global Clinical and Translational Research Institute, Bethesda, Maryland, USA; 7 Children’s Development Behavior Center, Third Affiliated Hospital of Sun Yat-sen University, Guangzhou, Guangdong, China; 8 Collaborative Innovation Center for Genetics and Development, Shanghai, China; 9 College of Life Science and Technology, Xinjiang University, Xinjiang, China

## Abstract

Autism spectrum disorder (ASD) describes a group of neurodevelopmental disorders with high heritability, although the underlying genetic determinants of ASDs remain largely unknown. Large-scale whole-genome studies of copy number variation in Han Chinese samples are still lacking. We performed a genome-wide copy number variation analysis of 343 ASD trios, 203 patients with sporadic cases and 988 controls in a Chinese population using Illumina genotyping platforms to identify CNVs and related genes that may contribute to ASD risk. We identified 32 rare CNVs larger than 1 Mb in 31 patients. ASD patients were found to carry a higher global burden of rare, large CNVs than controls. Recurrent *de novo* or case-private CNVs were found at 15q11-13, Xp22.3, 15q13.1–13.2, 3p26.3 and 2p12. The *de novo* 15q11–13 duplication was more prevalent in this Chinese population than in those with European ancestry. Several genes, including *GRAMD2* and *STAM*, were implicated as novel ASD risk genes when integrating whole-genome CNVs and whole-exome sequencing data. We also identified several CNVs that include known ASD genes (*SHANK3, CDH10, CSMD1*) or genes involved in nervous system development (*NYAP2, ST6GAL2, GRM6*). Besides, our study also implicated Contactins-NYAPs-WAVE1 pathway in ASD pathogenesis. Our findings identify ASD-related CNVs in a Chinese population and implicate novel ASD risk genes and related pathway for further study.

Autism spectrum disorders (ASDs) are a group of conditions characterized by impairments in social interaction and communication and by restricted, repetitive patterns of interest or behaviour^1^. The disorder presents clinically in the first 3 years of life. Its precise prevalence has proven difficult to estimate due to changing practices of diagnosis and ascertainment; rates have increased from no more than 5 per 10,000 individuals throughout the 1980 s to approximately 1 in 68 children today, according to a report from America’s CDC. Genetic epidemiology studies have indicated that ASDs have a high heritability^2^,^3^,^4^, suggesting a strong genetic basis for ASDs.

Evidence from different cases supports that *de novo* and rare chromosomal abnormalities, copy number variations (CNVs), single nucleotide variations (SNVs)/small insertion and deletions (InDels) and multiple common genetic variants contribute to the risk of ASD^5^. Dozens of *de novo* and rare inherited copy number variations (CNVs) have been found through large-cohort genome-wide studies that involved more than 5000 ASD families of primary European ancestry^6^,^7^,^8^,^9^,^10^,^11^,^12^,^13^. The most significant CNVs are 16p11.2 deletions and duplications, 7q11.23 duplications, 15q11–13 duplications, 1q21.1 duplications, 3q29 deletions, 22q11.2 deletions and duplications, deletions at *NRXN1* and deletions at *CDH13*^6^. These structural variants, many of which have large effects but are individually rare, together may account for approximately 4% of ASD cases^6^. Recently, whole-exome sequencing studies and candidate resequencing studies of large cohorts have also identified multiple genes with recurrent *de novo*, likely gene-disruptive (LGD) mutations in ASD patients, such as *CHD8, SYNGAP1, DYRK1A, ARID1B, SCN2A, DSCAM, ANK2, ADNP, POGZ, GRIN2B* and *CHD2*^14^,^15^,^16^,^17^,^18^,^19^,^20^. Common genetic variants have also been reported at 5p14.1 (*CDH10-CDH9*)^21^, 5p15.2 (*SEMA5A*)^22^, *MACROD2*^23^, *CNTNAP2*^24^ and 1p13.2 (*CSDE1, TRIM33*) loci^25^ through genome-wide association studies (GWAS). Together, these results suggest that ASD has a complex inheritance pattern; its genetic architecture and underlying mechanism are still largely unknown.

Although several large-cohort genome-wide CNV studies have been performed, few studies have investigated CNVs related to ASD in Chinese populations^26^,^27^. Previously, we performed a GWAS of autism in a Han Chinese population for common genetic variants associated with ASD^25^. Here, with additional samples, we present a genome-wide study in an attempt to identify CNVs that may contribute to the aetiology of ASD in the Han Chinese population. We found 32 rare, large CNVs longer than 1 Mb accounting for 5.68% of the total patients, of which five CNVs were recurrently identified in ASD patients.

## Results

### Burden of rare, large CNVs in ASD patients of a Chinese Han population

**Figure 1:**
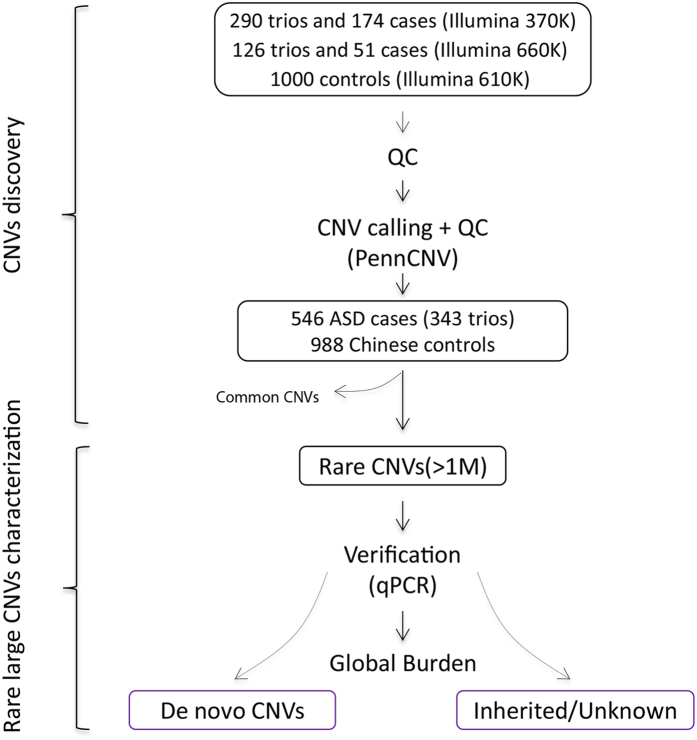
Pipeline of CNV discovery and analysis. ASD patients and parents were genotyped by an Illumina 370 K or 660 K BeadChip. Control subjects were genotyped using an Illumina 610 K BeadChip. CNV calling and quality control were performed using the PennCNV program. Rare, large CNVs (>1 Mb) were used for validation and analysis. The global burden was subsequently determined. *De novo* CNVs and inherited CNVs were characterized.

After strict quality control and CNV calling, 546 ASD subjects (343 trios) and 988 normal controls were analysed (Fig. 1). On average, 18 CNV calls per individual were made for samples genotyped using the HumanCNV370 BeadChip, 24.7 CNV calls for samples genotyped using the HumanCNV610 BeadChip and 357.3 CNV calls for samples genotyped using the HumanCNV660 BeadChip (the HumanCNV660 BeadChip includes many common CNV loci) (Figure S1). As we used low-density BeadChips and multiple genotyping platforms to perform the whole-genome genotyping, small CNV calls would not be confident and would be difficult to integrate from multiple platforms. Therefore, we only considered large CNVs (>1 Mb) in the analysis.

**Figure 2:**
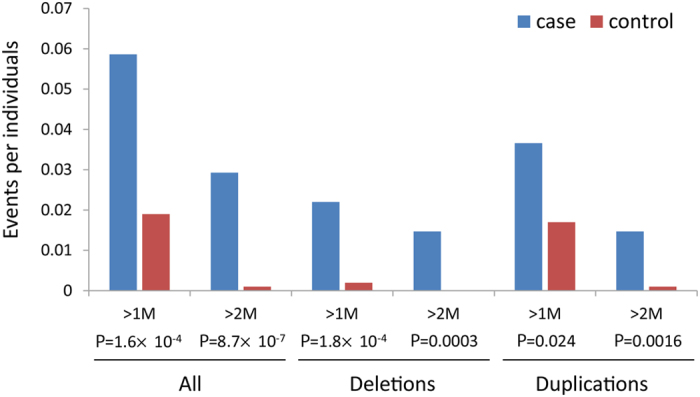
Burden analysis of rare, large CNVs in patients and controls. Deletions, duplications and the combined rate for all CNVs are shown. The CNV size was categorized as >1 Mb and >2 Mb. For each event type, the significance between patients and controls is given at the bottom.

We observed a higher CNV burden in patients with ASD. A total of 33 rare CNVs (<1%) larger than 1 Mb were identified in ASD patients by PennCNV, but one was not consistently validated (Figure S2). Finally, 32 rare CNVs were identified in 31 ASD probands (5.68% of the patients, Table 1), and 19 CNVs were identified in 19 control subjects (1.92% of the control subjects) (Table S1). The ASD patients had a significantly higher number of CNVs than the control subjects (odds ratio: 3.05, 95% CI: 1.66–5.74, p = 1.55E-04, Fisher’s exact test, Fig. 2, Table S2). The CNV burden was higher when considering CNVs larger than 2 Mb (odds ratio: 28.9, 95% CI: 4.47–1208.24, p = 8.7E-07, Fisher’s exact test). Both deletions and duplications contributed to the CNV burden. None of the 32 rare CNVs detected in this study were present in the control subjects. Among the 32 case-private CNVs, there were 16 *de novo* CNVs, 11 inherited CNVs, and five CNVs with unknown inheritance because of the absence of parental samples. Five CNVs were recurrently identified in 13 ASD patients (Table 1).

### Recurrent *de novo* or case-private CNVs

The 15q11–13 duplication was found to be one of the most common CNVs in the ASD patients. Of 32 CNVs, five *de novo* duplications were found at 15q11–13 (Table 1), which accounts for approximately 1% of the ASD patients in our study. We compared the frequency of this CNV with the Simons Simplex Collection (SSC) and Autism Genome Project (AGP) samples with primary European ancestry^6^,^7^ (Table S4). The incidence of 15q11–13 duplications was significantly higher in the Chinese population in our study (5-fold, p = 0.021, Fisher’s exact test). Among the five individuals with 15q11–13 duplications, two individuals carried three copy numbers of 15q11–13 respectively, and three individuals carried four copy numbers respectively. We then performed a karyotyping analysis using the blood samples of the patients and confirmed that the three incidences with four repeats were due to a partial tetrasomy of 15q (idic(15)) (Figure S3). A methylation assay confirmed that four of the duplications originated on maternally derived chromosomes (Table S3). The parent-of-origin of the last one is not confirmed because of the unsufficiency of the DNA quantity.

Several other recurrent CNVs were observed at four distinct chromosome regions. First, two deletions (3.5 Mb and 3.7 Mb) were found on chromosome Xp22.3, which includes the *NLGN4X* gene, in two ASD patients. The first proband (M8590) is a female with a *de novo* deletion; the other proband (M15199) is a male (hemizygous) whose mother carried the heterozygous deletion. Second, two deletions (1.39 Mb and 1.35 Mb) were found in 15q13.1–13.2, which includes the *APBA2* gene (Table 1, Figure S4). One of these deletions was *de novo*, whereas the other was maternally inherited. This is the first report of deletions involving the *APBA2* gene in ASD patients. Third, two duplications (1.9 Mb and 2.4 Mb) were detected on chromosome 3p26, which have been reported in our previous paper^28^. Both duplications disrupted the gene encoding Contactin, *CNTN4*, which has been implicated in developmental delays and ASDs. Finally, two duplications (1.1 Mb and 1.5 Mb) were detected on 2p12 in two ASD patients (Table 1). One was paternally inherited; the inheritance of the other is unknown. However, no known gene was found in this region.

### Integration of CNV and SNV data identified novel ASD candidate risk genes

In addition to the recurrent CNVs, 19 case-private CNVs were identified in individual ASD patients (Table 1). This included disruptions in four known ASD risk genes, *ARID1B, SHANK3, CDH10* and *CSMD1*. One large *de novo* deletion (21.9 Mb) at 6q24.3–6q27 contains the *ARID1B* gene, and one *de novo* deletion (2.6 Mb) at 22q13.3 includes *SHANK3* (Table 1). Two duplications disrupted genes encoding CUB and sushi domain-containing proteins (CSMDs), one of which (1.1 Mb) disrupted *CSMD1*, which has been implicated in ASD risk by a whole-exome sequencing study^29^. Notably, we identified another duplication (1.8 Mb) at 8q23.3 disrupting another gene encoding the CUB and sushi domain-containing protein *CSMD3* (Table 1). A maternally inherited deletion (2 Mb) at 5p14.2–14.1 disrupted a single gene, *CDH10* (Table 1), of which common variants have been implicated in ASD risk^21^.

**Figure 3:**
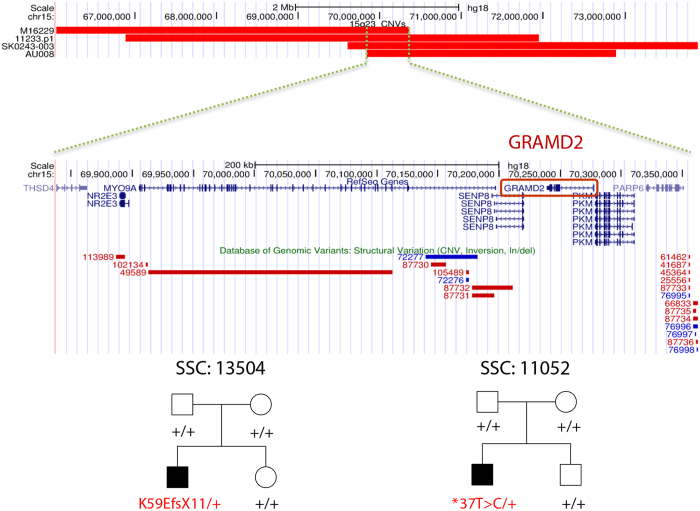
Convergence of *de novo* CNVs at 15q23 and *de novo* mutations of GRAMD2 within the overlapped region. Red bars indicate the chromosome locations of the four deletions identified in this study (M16229) and other studies (11233.p1, AU008, SK0243-003) (9, 13, 43). Two *de novo* mutations of GRAMD2 (a frameshift and an SNV in the 3’ UTR) were identified in two SSC simplex quad families (16). SSC family IDs and pedigree plots are presented at the bottom.

**Figure 4:**
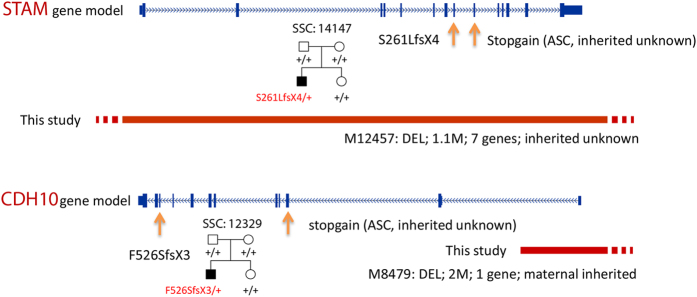
Convergence of rare private CNVs in this study and LGD mutations in SSC and ASC families or patients for *STAM* and *CHD10*. (**a**) Displayed for the *STAM* gene is a RefSeq gene model (larger ticks are exons), a loss-of-function deletion identified in this study, a *de novo* frameshift mutation (orange arrows) identified in an SSC quad simplex family and a nonsense mutation (orange arrows) identified in an ASC patient; (**b**). Displayed for the *CDH10* gene is a RefSeq gene model (larger ticks are exons), a disrupted deletion identified in this study and a *de novo* frameshift mutation (orange arrows) identified in SSC quad simplex families. SSC family IDs and pedigree plots are presented.

To identify novel potential ASD risk genes in these case-private CNVs (Table 1), we integrated *de novo* CNV data (Table S4) from large-cohort whole-genome CNV studies^6^,^7^,^8^,^9^,^10^,^11^,^13^ and SNV/InDel data (Table S5) from the SSC and Autism Sequencing Consortium (ASC) whole-exome sequencing studies^14^,^15^ to refine the CNV regions and search for potential *de novo* mutations or private LGD mutations of the genes represented by our case-private CNVs that were shorter than 5 Mb (n = 11). Finally, three published *de novo* CNVs were identified as overlapping with a *de novo* CNV (15q23) in this study (Fig. 3). The overlapped region includes only seven refSeq genes (Fig. 3). We analysed these genes in the SSC and ASC whole-exome sequencing data, and two *de novo* variants were identified in *GRAMD2* (Fig. 3). One of them is a frameshift mutation (p.K59EfsX11); the other is located in the 3’ UTR (c.*37 T > C). No other gene in this region was identified with a *de novo* mutation, and no *de novo* mutation of *GRAMD2* was identified in the unaffected SSC siblings. In addition, a *de novo* frameshift mutation (p. F526SfsX3) of *CDH10* was identified in an SSC simplex quad family, and an LGD mutation was identified in an ASC patient (Fig. 4). No *CDH10 de novo* mutation was identified in the unaffected SSC siblings or ASC controls. In addition, a *de novo* frameshift mutation (p.S261LfsX4) of *STAM* was identified in an SSC simplex quad family, and a missense *de novo* mutation was identified in an unaffected sibling (Fig. 4). A private LGD mutation of *STAM* was identified in an ASC patient. No LGD mutation was found in unaffected siblings and ASC controls.

## Discussion

We have performed a large-cohort genome-wide CNV study of ASD patients in a Chinese population to search for *de novo* and rare private CNVs that cause or contribute to the risk of ASD. Our study revealed the ASD-related CNVs (>1 Mb) in a Chinese population, which accounted for approximately 4% of the ASD cases. This study revealed multiple known ASD CNVs or genes and implicated novel ASD candidate CNVs or genes. Significantly, the 15q11–13 *de novo* duplication is more frequently found in Chinese samples compared with primary European samples. There are three plausible explanations for this finding: 1) the power of this study is low, and the finding will not be replicated with large populations; 2) the population analysed here is more severely affected than equivalent populations (SSC/AGP); and 3) there is a higher rate of mutation or penetrance at this locus in Han populations. Unfortunately, we do not have IQ data or other neurological examination data for the patients in our study and are therefore unable to address the second possibility. This is a limitation of our study. A larger detailed phenotyping cohort is needed to further explore this difference. Another limitation of this study is the low-density genotyping array and multiple platforms used. Because of this limitation, small CNVs (<1 Mb) were not investigated in this study. Another limitation is that the sample size is small considering the high genetic heterogeneity of ASD. Further genome-wide CNV studies using high-resolution approaches should be conducted in larger ASD cohorts from Chinese populations.

In this study, we identified known ASD risk CNVs or genes and implicated novel ASD risk CNVs or genes. The known ASD risk CNVs include those at 15q11–13 (*UBE3A, GABRB3*), Xp22.3–22.3 (*NLGN4X*), *CNTN4*, 22q13.31–13.33 (*SHANK3*) and *CDH10*. The overlapped region of 15 duplications on 15q11–13 identified from this study and other large-cohort studies include *UBE3A, GABRB3* and 11 other genes (Figure S5). The role of *UBE3A* has been clearly demonstrated by both functional studies and mouse model studies. *GABRB3* was recently implicated as another risk gene in this region^6^. *NLGN4X* has long been recognized as an ASD risk gene. Jamain *et al*. first identified *NLGN4X* as an ASD causative gene in 2003^30^. Subsequently, *NLGN4X de novo* and inherited mutations have been occasionally reported^31^,^32^. In total, 15 *NLGN4X* mutations were identified in the ASD patients in this study, including three missense mutations previously reported by our group (Figure S4). *CNTN4* has been implicated in development delays and ASDs. Up to this point, three studies, involving six patients or families with *CNTN4*-disruptive deletions or duplications, have been published^28^,^33^,^34^. The function of *APBA2* also supports the potential involvement of this gene in the pathogenesis of ASD. *APBA2* encodes a neuronal adaptor protein essential to synaptic transmission. It can form a complex that is able to couple synaptic vesicle exocytosis to neuronal cell adhesion. In addition, *APBA2* protein deficiency is associated with impaired social interaction in mice^35^. By integrating large-scale CNV and SNV data, our study also implicated several new ASD risk genes, including *GRAMD2* and *STAM*, which encode signal transducing adaptor molecules. The function of *GRAMD2* and *STAM* are still unclear. Further studies with both genetic and functional perspectives should be conducted to replicate the finding that these genes are involved in ASD risk.

**Figure 5:**
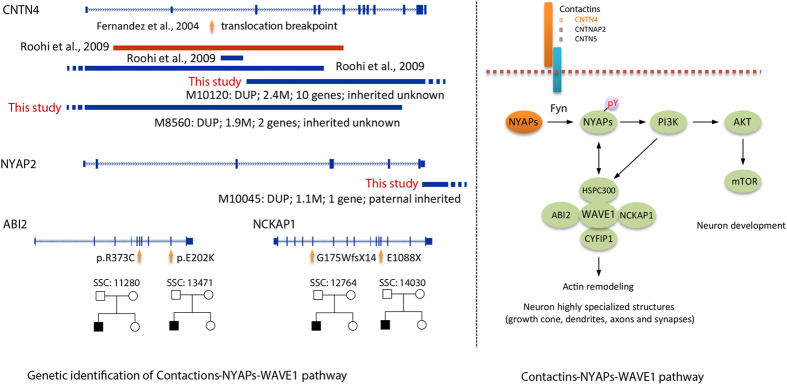
Genetic identification of genes involved in Contactins-NYAPs-WAVE1 pathway. Disruption of Contactin gene, *CNTN4*, was recurrently identified in this study and other studies. Case-private rare CNV disrupting *NYAP2* was also detected in this study. Red bars indicate deletions. The pedigree plots of SSC families with recurrent *de novo* mutations of *NCKAP1* and *ABI2* were presented. Orange arrows indicated the mutation location.

Several genes in the CNV regions that were not specifically described in the result section are also worthy of mention here. The first one is *NYAP2*, which is disrupted by a paternally inherited duplication (1.07 Mb) at 2q36.3 (Table 1). *NYAPs* are expressed predominantly in developing neurons. Three genes, *NYAP1, NYAP2, NYAP3*, encoding the Neuronal tYrosine-phosphorylated Adaptor for the PI3-kinase (NYAP) family of phosphoproteins, have been recognized. Interestingly, the *NYAP1* locus (7q22.1) and *NYAP3* locus (13q33.3) have suggestive linkage and association signals with ASD^21^,^36^. Upon stimulation with Contactins (such as *CNTN4, CNTNAP2* and *CNTN5*), NYAPs activate the downstream pathway including WAVE1 complex and PI3K-mTOR pathway^37^,^38^. The phosphalated NYAPs can active WAVE1 complex which function by interaction with several proteins, including *NCKAP1, ABI2, CYFIP2* and *HSP300*. Recently, recurrent *de novo* LGD mutations of *NCKAP1* were identified in ASD patients^15^. We further analyzed the SSC and ASC whole-exome sequencing data, two *de novo* missense mutations of *ABI2* were identified in two ASD probands (simplex quad families) but not for siblings (Fig. 5). *CYFIP1* was also implicated in ASD previous by both genetic and functional studies^39^,^40^. Therefore, disruption of Contactins-NYAPs-WAVE1 pathway (Fig. 5) appeared to be an important pathogenesis pathway in ASD etiology. In addition, there are two other genes worthy of mention. One is *GRM6* at 5q35.3, and the other is *ST6GAL2* at 2q12.2. *GRM6* encodes metabotropic glutamate receptor (mGluR). Interestingly, structural defects in the mGluR gene family interaction networks are significantly enriched in ASD patients^41^. In addition, common variants of *ST6GAL2* are associated with the risperidone response of schizophrenic patients^42^. Both of these genes are good candidates for further study.

## Conclusion

We performed the largest genome-wide copy number variation analysis of 546 ASD patients with 343 trios and 988 controls in a Han Chinese population. ASD patients were found to carry a higher global burden of rare, large CNVs. Recurrent *de novo* or case-private CNVs were found in five loci. The frequency of *de novo* 15q11–13 duplication was found to be significant higher compared with cohorts of European ancestry. In addition, several genes were implicated as novel ASD risk genes. Our findings identify ASD-related CNVs in a Chinese population, which could facilitate early disease testing and diagnosis and, most importantly, early intervention. Our study also implicated novel ASD risk genes, providing motivation for further functional and translational studies.

## Methods

### Study subjects

All the subjects who participated in this study completed informed consent before the original sample collection. ASD subjects were diagnosed independently by two experienced psychiatrists according to DSM-IV-TR criteria (American Psychiatric Association, 2000). The diagnostic procedure also included the assessment using a series of tools for neurological examination, mental status examination. A total of 406 ASD case-parent triad families (one affected offspring and two healthy parents) and 225 children with sporadic ASD were recruited for this study. The mean age of the patients was 5.065 years, and the mean age of onset was 2.5 years. An additional 1000 unrelated control subjects were also recruited. These control subjects had no history of ASDs or any other psychiatric diseases, nor did they have a familial history of psychiatric, neurological or autoimmune diseases. The mean age for the control subjects was 34.3 years. In summary, the study subjects include 406 ASD trios, 225 patients with sporadic cases and 1000 healthy controls of Chinese ancestry. This study was approved by the Institutional Review Board (IRB) of the State Key Laboratory of Medical Genetics, School of Life Sciences at Central South University, Changsha, Hunan, China and adhered to the tenets of the Declaration of Helsinki.

### Whole-genome genotyping

Genomic DNA was extracted from the whole blood for genome-wide genotyping. The DNA was extracted using a standard proteinase K digestion and phenol-chloroform method. All subject samples were genotyped using an Illumina BeadChip. The samples used in the initial GWAS of autism (290 autism trios and 174 autism cases) were genotyped using the Illumina HumanCNV370-Quad BeadChip including approximately 370 K SNPs, as demonstrated in our GWAS study^25^. The additional 126 trios and 51 sporadic cases were genotyped using the Illumina H*uman660W*-Quad BeadChip, which included approximately 660 K genetic variations. A cohort of 1,000 healthy controls was genotyped using the Illumina Human610-Quad BeadChip, which included approximately 610 K genetic variations. All SNPs in the HumanCNV370 BeadChip are covered in the Human610 BeadChip. All experiments were carried out in accordance with relevant guidelines.

### Quality control and CNV calling

Quality control was first performed based on the whole-genome genotyping data before CNV calling. We excluded individuals with likely poor DNA samples to avoid possible false positive CNVs. Individuals with a missing SNP call rate >2% were excluded. To reduce the noise of the genome-wide intensity signal, we only included samples whose standard deviation (SD) of normalized intensity was less than 0.3. Because wave artefacts roughly correlating with GC content resulting from the hybridization bias of low full-length DNA quantity are known to interfere with the accurate inference of copy number variations, only samples for which the correlation between the LRR and the wave model ranged between −0.2 < X < 0.4 were used in the CNV calling analysis. CNVs were called using the PennCNV algorithm^43^, which combines multiple sources of information, including the Log R Ratio (LRR) and B Allele Frequency (BAF) at each SNP marker, along with SNP spacing and population frequency of the B allele to generate CNV calls.

### CNV verification

The CNVs were validated by quantitative PCR (qPCR). Quantitative PCR validation was performed using the ABI Prism^TM^ 7900HT Sequence Detection System (Applied Biosystems, Foster City, CA). Three pairs of primers were selected from the start, middle and end of each CNV, separately. The sample was analysed in triplicate in a 10 μl reaction mixture (200 nM each primer, Maxima® SYBR Green/ROX qPCR Master Mix (2X) from Fermentas, and 5 or 10 ng of genomic DNA). The values were evaluated using System 7900 Software SDS2.3 (Applied Biosystems, CA). Further data analysis was performed using the qBase method. Reference genes, chosen from *COBL, GUSB*, and *SNCA*, were included based on the minimal coefficient of variation, and the data were then normalized by setting a normal control to a value of 1.

### Published CNV and exome sequencing data analysis

*De novo* CNV data were collected from six whole-genome CNV studies^6^,^7^,^8^,^9^,^10^,^11^,^13^ (Table S3). *De novo* SNV/InDel data were collected from the SSC and ASC whole-exome sequencing studies^14^,^15^ (Table S4). All CNVs and genes within our CNV regions of interest were analysed in the *de novo* CNV and SNV/InDel datasets both in cases and controls/unaffected siblings. We also examined ExAC data for the inferring variant deleteriousness and gene constraint of the implicated genes.

## Additional Information

**How to cite this article**: Guo, H. *et al*. Genome-wide copy number variation analysis in a Chinese autism spectrum disorder cohort. *Sci. Rep.* **7**, 44155; doi: 10.1038/srep44155 (2017).

**Publisher's note:** Springer Nature remains neutral with regard to jurisdictional claims in published maps and institutional affiliations.

## Supplementary Material

Supplementary Tables and Figures

## Figures and Tables

**Table 1:** Rare, large CNVs (>1 Mb) identified in this study.

Region^1^	Band	Length (bp)	CNV status^2^	Patient ID	Inheritance	Recurrent^3^	Candidate Gene^4^
***de novo***							
chr1:93416265-105648801	1p22.1-21.1	12,232,537	Del	M11409	*de novo*	N	56 genes (including OLFM3)
chr5:80564-8723219	5p15.33-15.2	8,642,656	Del	M8820	*de novo*	N	55 genes (including SLC9A3)
chr6:148602550-170469934	6q24.3-q27	21,867,385	Dup	M16084	*de novo*	N	126 genes (including ARID1B)
chr8:791912-16065839	8p23.3-p22	15,273,928	Del	M9118	*de novo*	N	102 genes (including RP1L1/XKR6)
chr15:19157192-26194101	15q11.2-q13.3	7,036,910	Dup = 4	M8145	*de novo*	Y(5)	142 gene (including UBE3A, GABRB3)
chr15:19767013-30690437	15q11.2-q13.3	10,923,425	Dup = 4	M15042	*de novo*	Y(5)	162 gene (including UBE3A, GABRB3)
chr15:20049770-30500698	15q11.2-q13.3	10,450,929	Dup = 4	M16079	*de novo*	Y(5)	155 gene (including UBE3A, GABRB3)
chr15:20306549-26219673	15q11.2-q13.1	5,913,125	Dup	M10117	*de novo*	Y(5)	119 gene (including UBE3A, GABRB3)
chr15:20314760-26208861	15q11.2-q13.1	5,894,101	Dup	M16147	*de novo*	Y(5)	109 gene (including UBE3A, GABRB3)
chr15:26762141-28153539	15q13.1-13.2	1,391,399	Del	M15147	*de novo*	Y(2)	6 genes (including APBA2)
chr15:66041595-70362563	15q23	4,320,969	Del	M16229	*de novo*	N	34 (including GRAMD2)
chr16:32090048-33240087	16p11.2	1,150,040	Dup	M8302	*de novo*	N	6 genes
chr20:55665989-62426157	20q13.31-13.33	6,760,169	Dup	M9118	*de novo*	N	96 genes
chr22:46871209-49498590	22q13.31-13.33	2,627,381	Del	M16087	*de novo*	N	40 genes (including SHANK3)
chrX:3944205-7480499	Xp22.33-22.31	3,536,295	Del	M8590	*de novo*	Y(2)	6 genes (including NLGN4X)
chrX:4482028-8177903	Xp22.32-22.31	3,695,876	Del	M15199	*de novo*	Y(2)	10 genes (including NLGN4X)
***inheritance unknown***							
chr2:81088888-82213957	2p12	1,125,070	Dup	M8725	unknown	Y(2)	.
chr3:1137041-2996663	3p26.3	1,859,623	Dup	M8560	unknown	Y(2)	2 genes (including CNTN4)
chr3:2610044-5012635	3p26.3-26.2	2,402,592	Dup	M10120	unknown	Y(2)	10 genes (including CNTN4)
chr8:2014561-3078991	8p23.3-23.2	1,064,431	Dup	M16201	unknown	N	2 genes (including CSMD1)
chr10:17559724-18663469	10q12.33	1,103,746	Dup	M12457	unknown	N	7 genes (including STAM)
***Inherited***							
chr2:106245033-107789242	2q12.2-12.3	1,544,210	Dup	M16053	paternal	N	5 genes (including ST6GAL2)
chr2:226225481-227298024	2q36.3	1,072,544	Dup	M10045	paternal	N	1 gene (including NYAP2)
chr2:81240319-82740470	2p12	1,500,152	Dup	M13360	paternal	Y(2)	.
chr4:189683236-190687299	4q35.2	1,004,064	Del	M11400	paternal	N	LINC01060
chr5:177795689-178865875	5q35.3	1,070,187	Dup	M10006	paternal	N	11 genes (including GRM6)
chr5:24638407-26660273	5p14.2-14.1	2,021,867	Del	M8479	maternal	N	1 gene (including CDH10)
chr5:69041250-70672298	5q13.2	1,631,049	Dup	M13395	maternal	N	12 genes
chr8:112622028-114424512	8q23.3	1,802,485	Dup	M12449	maternal	N	2 genes (including CSMD3)
chr15:26798209-28156445	15q13.1-13.2	1,358,237	Del	M12315	maternal	Y(2)	6 genes (including APBA2)
chr15:32021107-33298143	15q14	1,277,037	Dup	M11403	maternal	N	19 genes
chr17:14059029-15399033	17p12	1,340,005	Del	M8767	maternal	N	7 genes

Notes: 1. RefSeq version is hg18; 2. All Del is one copy number, all Dup is three copy numbers except M8145, M15042 and M16079, which are four copy numbers; 3. N represents private CNVs, Y represents recurrent CNVs, the numbers in the braces represent recurrent numbers; 4. All refSeq gene numbers, ASD risk genes or implicated ASD risk genes in the CNV regions are listed in the braces.

## References

[b1] Lai M. C., Lombardo M. V. & Baron-Cohen S. Autism. Lancet 383, 896–910 (2014). 24074734 10.1016/S0140-6736(13)61539-1

[b2] Bailey A. . Autism as a strongly genetic disorder: evidence from a British twin study. Psychological medicine 25, 63–77 (1995). 7792363 10.1017/s0033291700028099

[b3] Rosenberg R. E. . Characteristics and concordance of autism spectrum disorders among 277 twin pairs. Archives of pediatrics & adolescent medicine 163, 907–914 (2009). 19805709 10.1001/archpediatrics.2009.98

[b4] Constantino J. N., Zhang Y., Frazier T., Abbacchi A. M. & Law P. Sibling recurrence and the genetic epidemiology of autism. The American journal of psychiatry 167, 1349–1356 (2010). 20889652 10.1176/appi.ajp.2010.09101470 PMC2970737

[b5] Devlin B. & Scherer S. W. Genetic architecture in autism spectrum disorder. Current opinion in genetics & development 22, 229–237 (2012). 22463983 10.1016/j.gde.2012.03.002

[b6] Sanders S. J. . Insights into Autism Spectrum Disorder Genomic Architecture and Biology from 71 Risk Loci. Neuron 87, 1215–1233 (2015). 26402605 10.1016/j.neuron.2015.09.016 PMC4624267

[b7] Pinto D. . Convergence of genes and cellular pathways dysregulated in autism spectrum disorders. American journal of human genetics 94, 677–694 (2014). 24768552 10.1016/j.ajhg.2014.03.018 PMC4067558

[b8] Sebat J. . Strong association of de novo copy number mutations with autism. Science 316, 445–449 (2007). 17363630 10.1126/science.1138659 PMC2993504

[b9] Sanders S. J. . Multiple recurrent de novo CNVs, including duplications of the 7q11.23 Williams syndrome region, are strongly associated with autism. Neuron 70, 863–885 (2011). 21658581 10.1016/j.neuron.2011.05.002 PMC3939065

[b10] Levy D. . Rare de novo and transmitted copy-number variation in autistic spectrum disorders. Neuron 70, 886–897 (2011). 21658582 10.1016/j.neuron.2011.05.015

[b11] Pinto D. . Functional impact of global rare copy number variation in autism spectrum disorders. Nature 466, 368–372 (2010). 20531469 10.1038/nature09146 PMC3021798

[b12] Glessner J. T. . Autism genome-wide copy number variation reveals ubiquitin and neuronal genes. Nature 459, 569–573 (2009). 19404257 10.1038/nature07953 PMC2925224

[b13] Marshall C. R. . Structural variation of chromosomes in autism spectrum disorder. American journal of human genetics 82, 477–488 (2008). 18252227 10.1016/j.ajhg.2007.12.009 PMC2426913

[b14] De Rubeis S. . Synaptic, transcriptional and chromatin genes disrupted in autism. Nature 515, 209–215 (2014). 25363760 10.1038/nature13772 PMC4402723

[b15] Iossifov I. . The contribution of de novo coding mutations to autism spectrum disorder. Nature 515, 216–221 (2014). 25363768 10.1038/nature13908 PMC4313871

[b16] Neale B. M. . Patterns and rates of exonic de novo mutations in autism spectrum disorders. Nature 485, 242–245 (2012). 22495311 10.1038/nature11011 PMC3613847

[b17] Sanders S. J. . De novo mutations revealed by whole-exome sequencing are strongly associated with autism. Nature 485, 237–241 (2012). 22495306 10.1038/nature10945 PMC3667984

[b18] Iossifov I. . De novo gene disruptions in children on the autistic spectrum. Neuron 74, 285–299 (2012). 22542183 10.1016/j.neuron.2012.04.009 PMC3619976

[b19] O’Roak B. J. . Recurrent de novo mutations implicate novel genes underlying simplex autism risk. Nature communications 5, 5595 (2014). 10.1038/ncomms6595 PMC4249945 25418537

[b20] O’Roak B. J. . Multiplex targeted sequencing identifies recurrently mutated genes in autism spectrum disorders. Science 338, 1619–1622 (2012). 23160955 10.1126/science.1227764 PMC3528801

[b21] Wang K. . Common genetic variants on 5p14.1 associate with autism spectrum disorders. Nature 459, 528–533 (2009). 19404256 10.1038/nature07999 PMC2943511

[b22] Weiss L. A. . A genome-wide linkage and association scan reveals novel loci for autism. Nature 461, 802–808 (2009). 19812673 10.1038/nature08490 PMC2772655

[b23] Anney R. . A genome-wide scan for common alleles affecting risk for autism. Human molecular genetics 19, 4072–4082 (2010). 20663923 10.1093/hmg/ddq307 PMC2947401

[b24] Anney R. . Individual common variants exert weak effects on the risk for autism spectrum disorders. Human molecular genetics 21, 4781–4792 (2012). 22843504 10.1093/hmg/dds301 PMC3471395

[b25] Xia K. . Common genetic variants on 1p13.2 associate with risk of autism. Molecular psychiatry 19, 1212–1219 (2014). 24189344 10.1038/mp.2013.146

[b26] Gazzellone . Copy number variation in Han Chinese individuals with autism spectrum disorder. J Neurodev Disord. 6, 34 (2014) 25170348 10.1186/1866-1955-6-34 PMC4147384

[b27] Chong . Performance of chromosomal microarray for patients with intellectual disabilities/developmental delay, autism, and multiple congenital anomalies in a Chinese cohort. Mol Cytogenet 7, 34 (2014). 24926319 10.1186/1755-8166-7-34 PMC4055236

[b28] Guo H. . Disruption of Contactin 4 in two subjects with autism in Chinese population. Gene 505, 201–205 (2012). 22750301 10.1016/j.gene.2012.06.051

[b29] Krumm N. . Excess of rare, inherited truncating mutations in autism. Nature genetics 47, 582–588 (2015). 25961944 10.1038/ng.3303 PMC4449286

[b30] Jamain S. . Mutations of the X-linked genes encoding neuroligins NLGN3 and NLGN4 are associated with autism. Nature genetics 34, 27–29 (2003). 12669065 10.1038/ng1136 PMC1925054

[b31] Talebizadeh Z. . Novel splice isoforms for NLGN3 and NLGN4 with possible implications in autism. J Med Genet 43, e21 (2006). 16648374 10.1136/jmg.2005.036897 PMC2564526

[b32] Laumonnier F. . X-linked mental retardation and autism are associated with a mutation in the NLGN4 gene, a member of the neuroligin family. American journal of human genetics 74, 552–557 (2004). 14963808 10.1086/382137 PMC1182268

[b33] Roohi J. . Disruption of contactin 4 in three subjects with autism spectrum disorder. J Med Genet 46, 176–182 (2009). 18349135 10.1136/jmg.2008.057505 PMC2643049

[b34] Fernandez T. . Disruption of contactin 4 (CNTN4) results in developmental delay and other features of 3p deletion syndrome. American journal of human genetics 74, 1286–1293 (2004). 15106122 10.1086/421474 PMC1182094

[b35] Sano Y. . X11-like protein deficiency is associated with impaired conflict resolution in mice. The Journal of neuroscience: the official journal of the Society for Neuroscience 29, 5884–5896 (2009). 19420255 10.1523/JNEUROSCI.5756-08.2009 PMC6665217

[b36] International Molecular Genetic Study of Autism, C. A genomewide screen for autism: strong evidence for linkage to chromosomes 2q, 7q, and 16p. American journal of human genetics 69, 570–581 (2001). 11481586 10.1086/323264 PMC1235486

[b37] Yokoyama K. . NYAP: a phosphoprotein family that links PI3K to WAVE1 signalling in neurons. The EMBO journal. 30, 4739–4754 (2011). 21946561 10.1038/emboj.2011.348 PMC3243607

[b38] Mack T. G. & Eickholt B. J. New WAVEs in neuronal PI3K signalling. The EMBO journal. 30, 4693–4695 (2011). 22126817 10.1038/emboj.2011.405 PMC3242975

[b39] De Rubeis S. . CYFIP1 coordinates mRNA translation and cytoskeleton remodeling to ensure proper dendritic spine formation. Neuron. 79, 1169–1182 (2013). 24050404 10.1016/j.neuron.2013.06.039 PMC3781321

[b40] Pathania M. . The autism and schizophrenia associated gene CYFIP1 is critical for the maintenance of dendritic complexity and the stabilization of mature spines. Translational psychiatry. 4, e374 (2014). 24667445 10.1038/tp.2014.16 PMC3966042

[b41] Hadley D. . The impact of the metabotropic glutamate receptor and other gene family interaction networks on autism. Nature communications 5, 4074 (2014). 10.1038/ncomms5074 PMC4059929 24927284

[b42] Ikeda M. . Identification of novel candidate genes for treatment response to risperidone and susceptibility for schizophrenia: integrated analysis among pharmacogenomics, mouse expression, and genetic case-control association approaches. Biological psychiatry 67, 263–269 (2010). 19850283 10.1016/j.biopsych.2009.08.030

[b43] Wang K. . PennCNV: an integrated hidden Markov model designed for high-resolution copy number variation detection in whole-genome SNP genotyping data. Genome research 17, 1665–1674 (2007). 17921354 10.1101/gr.6861907 PMC2045149

