# Supplementary material for: Genome-wide copy number variation analysis in a Chinese autism spectrum disorder cohort
Source: Sci Rep. 2017 Mar 10;7:44155. doi: 10.1038/srep44155 (PMC5345089; doi:10.1038/srep44155)
Supplement: Supplementary Tables and Figures [file srep44155-s1.pdf]

Supplemental Information

Genome-wide copy number variation analysis in a Chinese autism spectrum disorder cohort

Hui Guo, Yu Peng, Zhengmao Hu, Ying Li, Guanglei Xun, Jianjun Ou, Liangdan Sun, Zhimin Xiong, Yanling Liu, Tianyun Wang, Jingjing Chen, Lu Xia, Ting Bai, Yidong Shen, Qi Tian, Yiqiao Hu, Lu Shen, Rongjuan Zhao, Xuejun Zhang, Fengyu Zhang, Jingping Zhao, Xiaobing Zou & Kun Xia

Table S1. All rare large CNVs (>1M) identified in autism cases and controls

Region	numSNP	Length (bp)	CNVstatus	IID	Inheritance	phenotypes
chr5:177795689-178865875	5q35.3	1,070,187	Dup	M10006	paternal	case
chr2:226225481-227298024	2q36.3	1,072,544	Dup	M10045	paternal	case
chr15:20306549-26219673	806	5,913,125	Dup	M10117	de novo	case
chr3:2610044-5012635	514	2,402,592	Dup	M10120	unknown	case
chr4:189683236-190687299	185	1,004,064	Del	M11400	paternal	case
chr15:32021107-33298143	173	1,277,037	Dup	M11403	maternal	case
chr1:93416265-105648801	1389	12,232,537	Del	M11409	de novo	case
chr15:26798209-28156445	173	1,358,237	Del	M12315	maternal	case
chr5:102946704-104118535	172	1,171,832	Dup	M12412	unknown	case
chr8:112622028-114424512	153	1,802,485	Dup	M12449	maternal	case
chr10:17559724-18663469	193	1,103,746	Dup	M12457	unknown	case
chr2:81240319-82740470	180	1,500,152	Dup	M13360	paternal	case
chr5:69041250-70672298	267	1,631,049	Dup	M13395	maternal	case
chr15:19767013-30690437	2903	10,923,425	Dup	M15042	de novo	case
chr15:26762141-28153539	335	1,391,399	Del	M15147	de novo	case
chrX:4482028-8177903	503	3,695,876	Del	M15199	de novo	case
chr2:106245033-107789242	350	1,544,210	Dup	M16053	paternal	case
chr15:20049770-30500698	2776	10,450,929	Dup	M16079	de novo	case
chr6:148602550-170469934	7052	21,867,385	Dup	M16084	de novo	case
chr22:46871209-49498590	1062	2,627,381	Del	M16087	de novo	case
chr2:203007467-204075021	150	1,067,555	Del	M16134	unknown	case
chr15:20314760-26208861	1721	5,894,101	Dup	M16147	de novo	case
chr8:2014561-3078991	631	1,064,431	Dup	M16201	unknown	case
chr15:66041595-70362563	1031	4,320,969	Del	M16229	de novo	case
chr15:19157192-26194101	906	7,036,910	Dup	M8145	de novo	case
chr16:32090048-33240087	206	1,150,040	Dup	M8302	de novo	case
chr5:24638407-26660273	220	2,021,867	Del	M8479	maternal	case
chr3:1137041-2996663	501	1,859,623	Dup	M8560	unknown	case
chrX:3944205-7480499	313	3,536,295	Del	M8590	de novo	case
chr2:81088888-82213957	89	1,125,070	Dup	M8725	unknown	case
chr17:14059029-15399033	17p12	1,340,005	Del	M8767	maternal	case
chr5:80564-8723219	1436	8,642,656	Del	M8820	de novo	case
chr8:791912-16065839	3204	15,273,928	Del	M9118	de novo	case
chr20:55665989-62426157	1156	6,760,169	Dup	M9118	de novo	case
chr1:236170124-237444685	344	1,274,562	Del	PY2819	unknown	control
chr2:212497140-213573669	309	1,076,530	Dup	PY2755	unknown	control
chr2:33878579-34887500	278	1,008,922	Dup	PY4121	unknown	control
chr2:56782143-58336150	291	1,554,008	Dup	PY2774	unknown	control
chr2:78304452-79943327	495	1,638,876	Dup	PY4156	unknown	control
chr3:74600103-75738560	171	1,138,458	Dup	PY1296	unknown	control
chr4:179102293-180475342	261	1,373,050	Dup	PY4091	unknown	control
chr7:9749547-10924197	298	1,174,651	Dup	PY1181	unknown	control
chr7:98459808-99464231	172	1,004,424	Dup	PY1118	unknown	control
chr8:3887015-6054003	1456	2,166,989	Dup	PY2614	unknown	control
chr10:2231511-3261675	390	1,030,165	Dup	PY1396	unknown	control
chr10:6548383-7619095	389	1,070,713	Dup	PY2991	unknown	control
chr13:69393269-70497303	306	1,104,035	Dup	PY1156	unknown	control
chr14:43983784-45341456	197	1,357,673	Dup	PY2555	unknown	control
chr15:29018547-30302218	294	1,283,672	Dup	PY2562	unknown	control
chr16:71630790-72882363	399	1,251,574	Del	PY2820	unknown	control
chr17:14030694-15411904	481	1,381,211	Del	PY2576	unknown	control
chr18:34087299-35222878	232	1,135,580	Del	PY2746	unknown	control
chr22:22143258-23307901	277	1,164,644	Del	PY4109	unknown	control

Table S2. Burden analysis of rare large CNVs (>1M) in cases and controls (Fisher's exact test).

Type	CNVs Size	CNVs Num in Cases	CNVs Num in controls	P	OR	95% CI
All	>1M	32	19	1.55E-04	3.05	1.66-5.74
	>2M	16	1	8.70E-07	28.9	4.47-1208.24
Deletion	>1M	12	2	1.83E-04	10.84	2.40-100.12
	>2M	8	0	0.00027	Inf	3.07-Inf
Duplication	>1M	20	17	0.02438	2.13	1.05-4.36
	>2M	8	1	0.00164	14.45	1.93-640.97

Table S3. The results of methylation assay of 4 samples (M8145, M15042, M16079, M1017) with 15q11-13 duplication.

sampleID	TA-cloneID	CpG island methylation status, + methylated, - unmethylated																							
		1	2	3	4	5	6	7	8	9	10	11	12	13	14	15	16	17	18	19	20	21	22	23	24
M8145	1	-	-	-	-	-	-	-	-	-	-	-	-	-	-	-	-	-	-	-	-	-	-	-	-
	2	+	+	+	+	+	+	+	+	-	+	+	+	+	+	+	+	+	+	+	+	+	+	+	+
	3	+	+	+	+	+	+	+	+	-	+	+	+	+	+	+	+	+	+	+	+	+	+	-	-
	4	+	+	+	+	+	+	+	+	-	+	+	+	+	+	+	+	+	+	+	+	+	+	+	+
	5	+	+	+	+	+	+	+	+	-	+	+	+	+	+	+	+	+	+	+	+	+	-	+	+
	6	+	+	+	+	+	+	+	+	-	+	+	+	+	+	+	+	+	+	+	+	+	+	-	-
	7	+	+	+	+	+	+	+	+	-	+	+	+	+	+	+	+	+	+	+	+	+	+	-	-
	8	-	-	-	-	-	-	-	-	-	+	+	+	+	+	+	+	+	+	+	+	-	-	-	-
	9	+	+	+	+	+	+	+	+	-	+	+	+	+	+	+	+	+	+	+	+	+	-	-	-
	10	+	+	+	+	+	+	+	+	-	+	+	+	+	+	+	+	+	+	+	+	+	-	-	-
M15042	1	+	+	+	+	+	+	+	+	+	+	+	+	+	+	+	+	+	+	+	+	+	+	+	+
	2	+	+	+	+	+	+	+	-	+	+	+	+	+	+	+	+	+	+	+	+	+	+	+	+
	3	+	+	+	+	+	+	+	+	+	+	+	+	+	+	+	+	+	+	+	+	-	+	+	+
	4	+	+	+	-	+	+	+	+	+	+	-	-	-	-	-	-	-	-	-	-	-	-	+	+
	5	+	+	+	-	+	+	+	+	+	+	+	+	-	+	+	+	+	+	-	+	+	-	-	-
	6	+	+	+	+	+	+	+	+	+	+	+	+	+	+	+	+	+	+	+	+	+	+	+	+
	7	-	-	-	-	-	-	-	-	-	-	-	-	-	-	-	-	-	-	-	-	-	-	+	+
	8	+	+	+	-	+	+	+	+	+	+	+	+	+	+	+	+	+	+	+	+	+	+	+	+
	9	+	+	+	+	+	+	+	-	+	+	+	+	+	+	+	+	+	+	+	+	+	+	+	+
	10	-	-	-	-	-	-	-	-	-	-	-	-	-	-	-	-	-	-	-	-	-	-	+	+

M16079	1	+	+	+	+	+	+	+	+	+	+	+	+	+	+	+	+	+	+	+	-	+	+	+	+
	2	+	+	+	+	+	+	+	+	+	+	+	+	+	+	+	+	+	+	+	-	+	+	+	+
	3	+	+	+	+	+	+	+	+	+	+	+	+	+	+	+	+	+	+	+	+	+	+	+	+
	4	+	+	+	+	+	+	+	+	+	+	+	+	+	+	+	+	+	+	+	+	+	+	+	+
	5	+	+	+	+	+	+	+	+	+	+	+	+	+	+	+	+	+	+	+	+	+	+	+	+
	6	+	+	+	+	+	+	+	+	+	+	+	+	+	+	+	+	+	+	+	+	+	+	+	+
	7	+	+	+	+	+	+	+	+	+	+	+	+	+	+	+	+	+	+	+	+	+	+	+	+
	8	+	-	+	+	+	+	+	+	+	+	+	+	+	+	+	+	+	+	+	-	+	+	+	+
	9	+	+	+	+	+	+	+	+	+	+	+	+	+	+	+	+	+	+	+	+	+	+	+	+
	10	+	+	+	+	+	+	+	+	+	+	+	+	+	+	+	+	+	+	+	+	+	+	+	+
M10117	7	+	+	+	+	+	+	+	+	+	+	+	+	+	+	+	+	+	+	+	+	+	+	+	+
	5	+	+	+	+	+	+	+	+	+	+	+	+	+	+	+	+	+	+	+	+	+	+	+	+
	8	+	+	+	+	+	+	+	+	+	+	+	+	+	+	+	+	+	+	+	+	+	+	+	+
	9	+	+	+	+	+	+	+	+	+	+	+	+	+	+	+	+	+	+	+	+	+	+	+	+
	3	+	+	+	+	+	+	+	+	+	+	+	+	+	+	+	+	+	+	+	+	+	+	+	+
	1	+	+	+	+	+	+	+	+	+	+	+	+	+	+	+	+	+	+	+	+	+	+	+	+
	4	+	+	+	+	+	+	+	+	+	+	+	+	+	+	+	+	+	+	+	+	+	+	+	+
	2	+	+	+	+	+	+	+	+	+	+	+	+	+	+	+	+	+	+	+	+	+	+	+	+
	6	+	+	+	+	+	+	+	+	+	+	+	+	+	+	+	+	+	+	+	+	+	+	+	+
	10	-	-	-	-	-	-	-	-	-	-	-	-	-	-	-	-	-	-	-	-	-	-	-	-

Notes: Methylation status of M266 was assayed for 10 TA clones/sample. M266 sequence (24 CpG islands):

CCAGGTCATTCCGGTGAGGGAGGGAGCTGGGACCCCTGCACTGCGGCAAACAAGCACGCCTGCGCGGCCGCAGAGGCAGGCTGGCGCGCATGCTCAGGCGGGGATGTGTGCGAAGCCTGCCGCTGCTGCAGCGAGTCTG
GCGCAGAGTGGAGCGGCCGCCGGAGATGCCTGACGCATCTGTCTGAGGAGCGGTCAGTGACGCGATGGAGCGGGCAAGGTCAGCTGTGCCGGTGGCTTCTCTCAAGAGACAGCCTGGGGAGCGGCCACTTTTATTCATC
AGATATTCCAAGTTTTTAGGACTTGGAGTACTGAATAAACGGAATTTGGGCCCTAAAGTCCTTTGTTCTGGAGAACCAGATCCGGAATGTTCAGAGGCTTGCTGTTGTGC

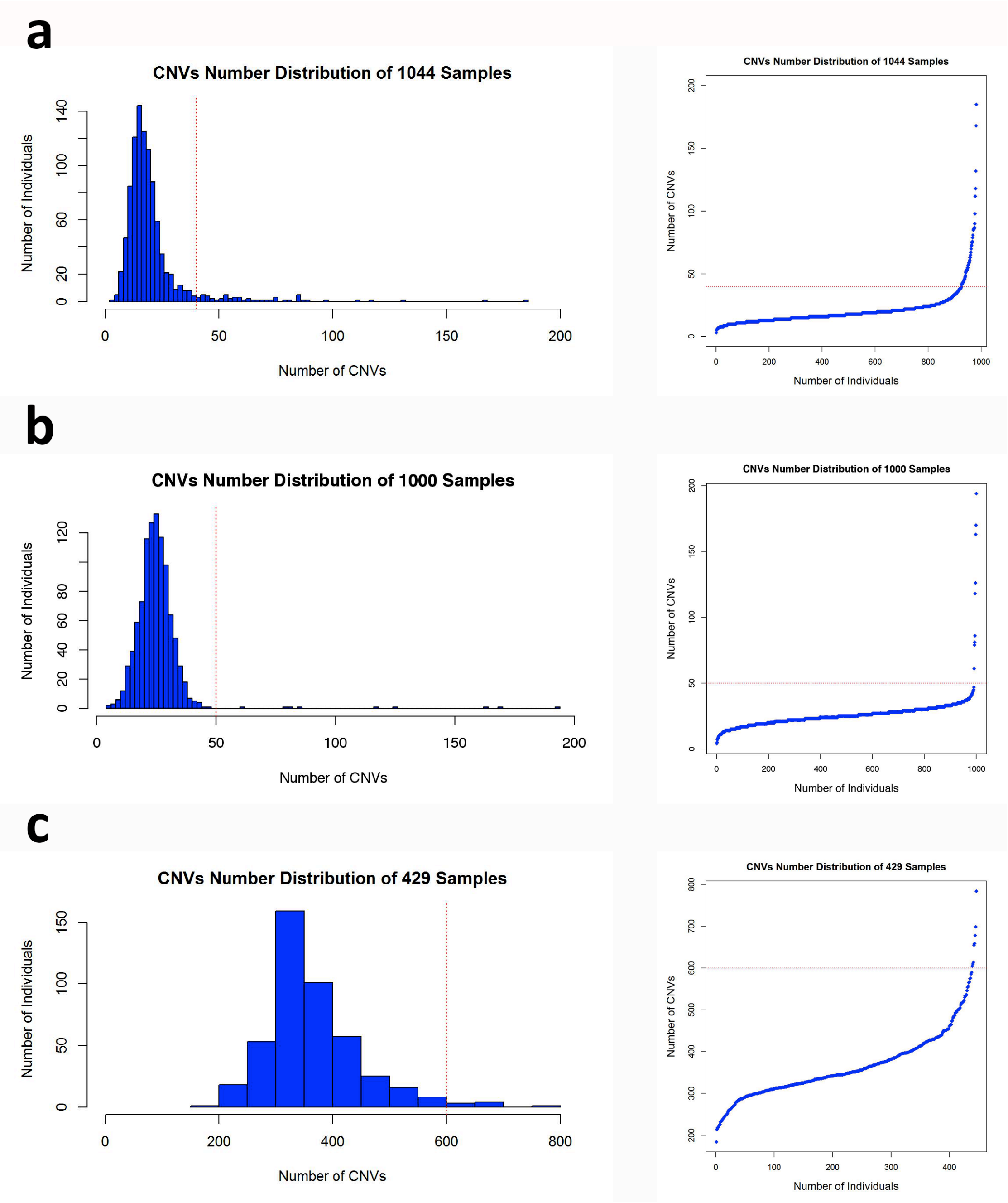

Figure S1. The distribution of CNV calls per individual. a. The distrubutionof CNV calls of 1044 samples (290 trios and 174 cases) genotyped by Illumina HumanCNV370-Quad BeadChip; b. The distrubutionof CNV calls of 1000 control samples genotyped by Illumina Human610-Quad BeadChip; c. The distrubutionof CNV calls of 429 samples (126 trios and 51 cases) genotyped by Illumina Human660W-Quad BeadChip.

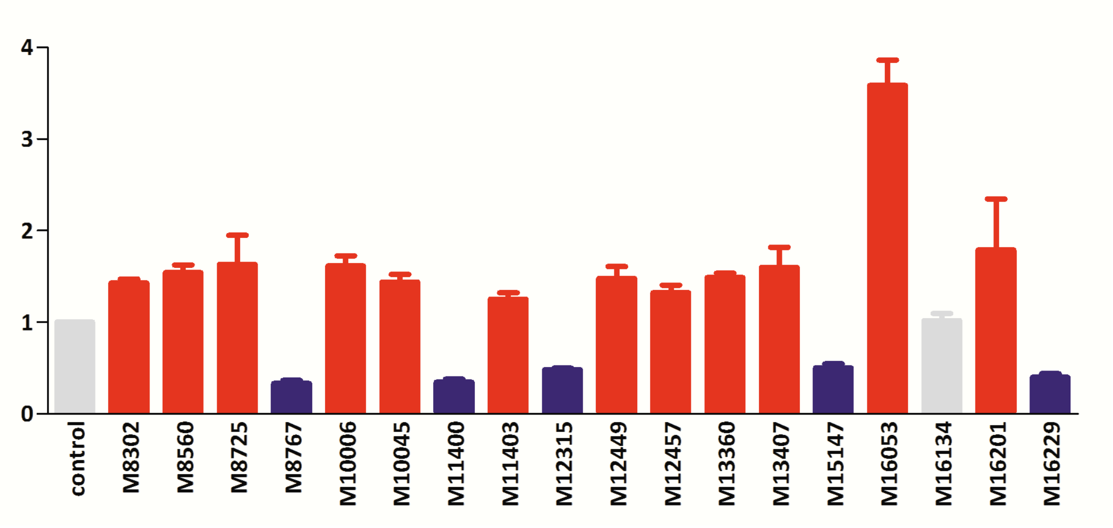

Figure S2. qPCR validation of CNVs with size less than 5M.

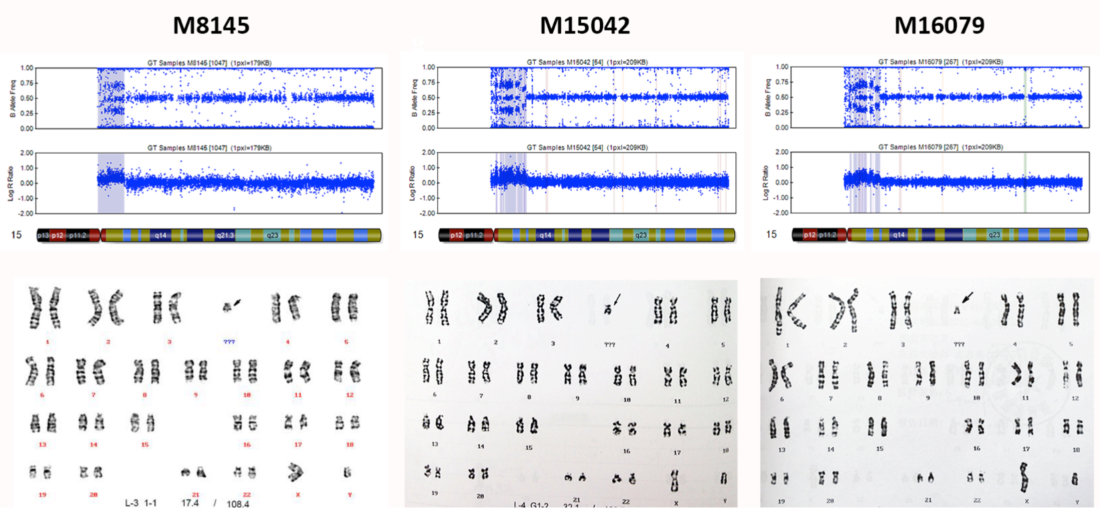

Figure S3. BeadChip scatter plots (top) and Karyotype plots (down) of three cases with idic(15).

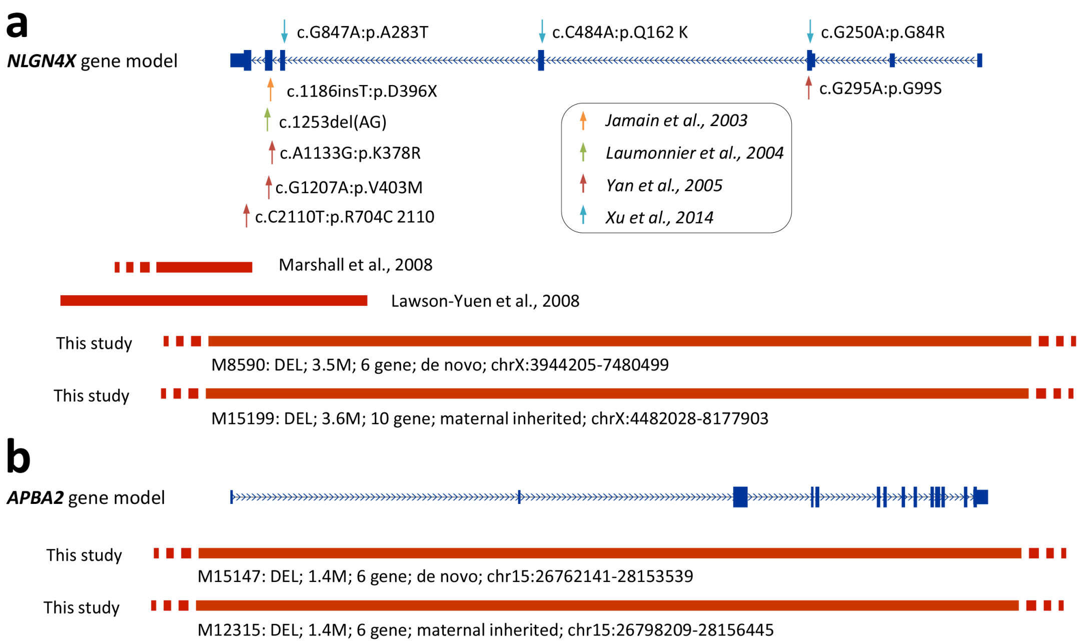

Figure S4: a. Convergence of de novo or rare private CNVs and other previous reported de novo mutations of NLGN4X. b. Two deletions (1 de novo, 1 inherited) invovled APBA2 gene identified in 2 autism cases in this study.

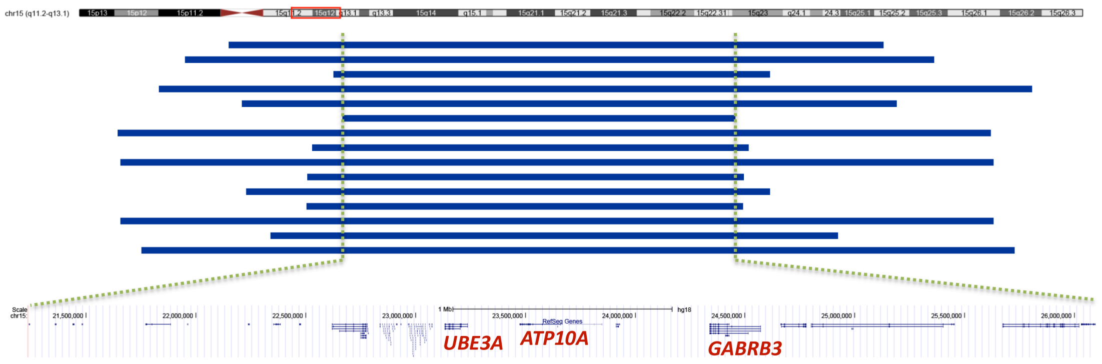

Figure S5. The smallest overlap region of 15q11-13 duplications identified in this study and previous large scale studies. The overlap region contains 3 important genes in neuron development including UBE3A, GABRB3 and ATP10A. The role of UBE3A has been clearly demonstrated in the risk of autism both from functional studies and mouse model studies. GABBA3 was appeared to be another risk gene in this region identified recently by exome sequencing study (1).

References:

1. Sanders SJ, He X, Willsey AJ, Ercan-Sencicek AG, Samocha KE, Cicek AE, et al. (2015): Insights into Autism Spectrum Disorder Genomic Architecture and Biology from 71 Risk Loci. *Neuron*. 87:1215-1233.
